# Supplementary material for: HEalth And Dementia outcomes following Traumatic Brain Injury (HEAD-TBI): protocol for a retrospective cohort study
Source: BMJ Open. 2023 Jul 25;13(7):e073726. doi: 10.1136/bmjopen-2023-073726 (PMC10373748; doi:10.1136/bmjopen-2023-073726)
Supplement: Author's manuscript [file bmjopen-2023-073726.draft_revisions.pdf]

BMJ Open

BMJ Open is committed to open peer review. As part of this commitment we make the peer review history of every article we publish publicly available.

When an article is published we post the peer reviewers' comments and the authors' responses online. We also post the versions of the paper that were used during peer review. These are the versions that the peer review comments apply to.

The versions of the paper that follow are the versions that were submitted during the peer review process. They are not the versions of record or the final published versions. They should not be cited or distributed as the published version of this manuscript.

BMJ Open is an open access journal and the full, final, typeset and author-corrected version of record of the manuscript is available on our site with no access controls, subscription charges or pay-per-view fees (http://bmjopen.bmj.com).

If you have any questions on BMJ Open's open peer review process please email info.bmjopen@bmj.com

BMJ Open

HEalth And Dementia outcomes following Traumatic Brain Injury (HEAD-TBI): protocol for a retrospective cohort study

Journal:	*BMJ Open*
Manuscript ID	bmjopen-2023-073726
Article Type:	Protocol
Date Submitted by the Author:	14-Mar-2023
Complete List of Authors:	Russell, Emma; University of Glasgow, School of Psychology and Neuroscience<br>Lyall, Donald; University of Glasgow, School of Health and Wellbeing<br>Stewart, William; University of Glasgow, School of Psychology and Neuroscience ; NHS Greater Glasgow and Clyde, Neuropathology Research Laboratory
Keywords:	Dementia < NEUROLOGY, Neuropathology < NEUROLOGY, PUBLIC HEALTH

SCHOLARONE™
Manuscripts

HEalth And Dementia outcomes following Traumatic Brain Injury (HEAD-TBI): protocol for a retrospective cohort study

Emma R Russell[1], Donald M Lyall[2], William Stewart[1,3]

[1]School of Psychology and Neuroscience, University of Glasgow, Glasgow G12 8QQ, UK
[2]School of Health and Wellbeing, University of Glasgow, Glasgow G12 8RZ, UK
[3]Department of Neuropathology, Queen Elizabeth University Hospital, Glasgow G51 4TF, UK

Corresponding Author: Dr Emma Russell, PhD
School of Psychology and Neuroscience
University of Glasgow
Glasgow G12 8QQ, UK

Email: emma.russell@glasgow.ac.uk
Tel: +44(0)141 354 9445

Word Count: 2,400

For peer review only - http://bmjopen.bmj.com/site/about/guidelines.xhtml

Abstract

Background It is estimated that by 2050 the global incidence of dementia will have exceeded 152million. At present there are no effective therapies for dementia, with a focus in research now turning to strategies for disease prevention. Traumatic brain injury (TBI) is recognised as a major risk factor for dementia; estimated to be responsible for at least 3% of cases in the community. However, adverse health outcomes after TBI are not restricted to dementia. A wide range of conditions are documented among TBI survivors, many of which also increase dementia risk. "*HEalth And Dementia outcomes following Traumatic Brain Injury (HEAD-TBI)*" is a study aiming to explore the hypothesis that increased dementia risk following TBI reflects both the direct effect of the injury on the brain and the indirect effects of wider, adverse health outcomes associated with TBI which, in turn, increase dementia risk.

Methods and analysis Comprehensive electronic medical and death certification records will be analysed for individuals with a documented history of TBI, compared with those of a matched general population control cohort with no documented TBI exposure. Cox proportional hazard regression models will be run to compare outcomes. Furthermore, existing diagnostic imaging and radiological reports for the cohort will be analysed to identify evidence of specific white matter abnormalities in TBI exposed individuals and their controls, and establish their potential diagnostic utility.

Ethics and dissemination Approvals for the study have been obtained from the University of Glasgow College of Medical, Veterinary, and Life Sciences Research Ethics Committee (Project Number 200220038) and from National Health Service Scotland's Public Benefits and Privacy Panel (Application 2122-0224).

For peer review only - http://bmjopen.bmj.com/site/about/guidelines.xhtml

Strengths and limitations of this study

- HEAD-TBI will provide novel insights into the relationship between traumatic brain injury and neurodegenerative disease risk with potential to inform strategies to identify those at greatest risk of adverse outcome and possibilities for intervention to mitigate risk.

- Utilising a comprehensive dataset of individuals with a history of traumatic brain injury compared with matched population controls – matched on sex, year of birth, and socioeconomic status – minimises risks of bias.

- It is known that there are limitations in the granularity of the data – brain injury data will be coded in accordance with the International Classification of Diseases, we will not have access to self-reported information or GP/hospital notes.

- Errors in health record coding are widely recognised, but are assumed to be consistent between our cohort of interest (those with a history of traumatic brain injury) and their matched population controls.

- The NHS was not founded until 1948, and we therefore may have less complete medical records for individuals born in the early 1900's.

For peer review only

For peer review only - http://bmjopen.bmj.com/site/about/guidelines.xhtml

Background

Over a quarter of a million people in the UK are living with a dementia diagnosis, with an annual cost to the economy of around £34billion[1]. Despite decades of research, we have no effective therapies for dementia. In part, this is a consequence of the disease having been active for many years by the time a diagnosis is made, by which time the neurodegenerative process is both established and irreversible. Attention, therefore, has begun to focus on developing strategies to prevent or delay the onset of disease. Reflecting this, dementia prevention is regarded as a major public health priority by the World Health Organization[2]. Further, the 2021 Alzheimer's Innovation Readiness Index, co-authored by Alzheimer's Disease International (ADI) and the Global Coalition on Aging (GCOA), calls for national campaigns to address dementia risk factors and for investment in research to better understand their contribution to disease[3].

The 2020 Lancet Commission on Dementia, Intervention and Care identifies 12 potentially modifiable risk factors for dementia[4]. Among these, TBI is estimated to contribute to in excess of 3% of dementias in the community. Importantly, whereas for many, if not all other dementia risk factors, the temporal relationship between risk of exposure and onset of neurodegenerative process is unknown, for TBI the time of injury is known (because it is a discrete, single 'event'). As such, studies in patients with TBI provide unique opportunity to track the development of disease over time, with potential to identify novel strategies for early diagnosis and intervention. However, while the association between TBI and neurodegenerative disease risk is recognised, this is not the only adverse health outcome associated with TBI[5,6]. In addition to increased risk of neurodegenerative disease, individuals with history of TBI are also recognised as having earlier mortality than expected, with increased risk of many common diseases such as cardiovascular disease, cancer, and respiratory diseases[7]. As such, increased risk of neurodegenerative disease in those surviving TBI might reflect the direct effect of the injury to the brain and/or a consequence of wider adverse health outcomes contributing to increased dementia risk.

HEalth And Dementia outcomes following Traumatic Brain Injury (HEAD-TBI) is specifically designed to assess the complex interaction between TBI and lifelong health outcomes contributing to dementia risk following injury. In so doing, HEAD-TBI

For peer review only - http://bmjopen.bmj.com/site/about/guidelines.xhtml

will provide novel insights into the relationship between TBI and neurodegenerative disease risk with potential to inform strategies to identify those at greatest risk of adverse outcome and possibilities for intervention to mitigate risk. In addition, by providing unique opportunity to study trajectory of disease from initiation to outcome, HEAD-TBI will have potential wider application to research in non-TBI associated dementias. The study will run for a duration of 3 years, during October 2022 to October 2025.

Aims

The specific aims of HEAD-TBI are to:

- explore the risk of dementia and wider neurodegenerative diseases in individuals with a history of TBI compared to matched general population controls.
- compare causes of death and wider health outcomes, including mental health, in individuals with a history of TBI compared to matched general population controls.
- assess the relationship between structural brain imaging differences and risk of dementia following TBI.

Methods and analysis

Using established protocols that have been proven highly successful in addressing similar research aims in other populations of interest[8,9], we now propose conducting a retrospective cohort study looking at a wide range of physical and mental health outcomes, including neurodegenerative disease, in individuals exposed to traumatic brain injury, compared to an age, sex, and social deprivation matched general population comparison group. Prior to study start, appropriate ethical approval has been sought from the University of Glasgow College of Medical, Veterinary, and Life Sciences Ethics committee, and National Health Service (NHS) Scotland's Public Benefit and Privacy Panel (PBPP) for Health and Social Care. All health records data will be anonymised to researchers, thus participant level consent will not be required for the purposes of this study.

Health record interrogation

Individuals with a documented history of TBI will be our cohort of interest, and will be identified from records held by the electronic data research and innovation service (eDRIS), who are part of Public Health Scotland, and hold a wide range of health

For peer review only - http://bmjopen.bmj.com/site/about/guidelines.xhtml

information, including prescription information and hospital attendance, as well as death certification data for residents in Scotland. Individuals who have prior TBI recorded (coded as international classification of disease 9/10) in their medical records (namely hospital inpatient and outpatient attendance SMR00/SMR01) will be selected. Individuals will be considered to have a documented TBI if their hospital inpatient or outpatient records contain one of the ICD9/ICD10 codes shown in tables 1 & 2. A wide range of TBI severities are listed in the chosen codes, ranging from those with a history of mild TBI such as concussion, to those with more moderate-to-severe brain injuries. This will enable for broad analyses covering all TBI outcomes, to more stratified analyses investigating lifelong health outcomes following certain types or severities of injury.

Table 1: ICD10 codes for traumatic brain injury

S06.0-S06.9	Concussion<br>Traumatic cerebral oedema<br>Diffuse brain injury<br>Focal brain injury<br>Epidural haemorrhage<br>Traumatic subdural haemorrhage<br>Traumatic subarachnoid haemorrhage<br>Intracranial injury with prolonged coma<br>Other intracranial injuries<br>Intracranial injury, unspecified
S07.1, S07.8, S07.9	Crushing injury of skull<br>Crushing injury of other parts of the head<br>Crushing injury of head, unspecified
S09.7-S09.9	Multiple injuries of head<br>Other specified injuries of head<br>Unspecified injury of head

Table 2: ICD9 codes for traumatic brain injury

850.1-850.5, 850.9<br>851.0-854.1	Intracranial injury, including concussion, confusion, laceration, and haemorrhage
959.01	Head injury, unspecified

Matched population controls for the study cohort will be selected on a 3:1 basis, i.e. for every one individual with exposure to traumatic brain injury, three matched controls will be selected. This aims to minimise any bias that may be present, and to increase study power, which can aid the detection of a statistically significant difference in outcome variables. These matched population controls will also be identified via the

For peer review only - http://bmjopen.bmj.com/site/about/guidelines.xhtml

electronic Data Research and Innovation Service (eDRIS) of Information Services Division of NHS Scotland. Controls will be matched on year of birth, sex, and socioeconomic status. Socioeconomic status is taken from last known postcode, from which area-based socioeconomic deprivation can be calculated using the Scottish Index of Multiple Deprivation (SIMD) 2020 - which takes into account income, employment, health, education, housing and crime rates of that area. The SIMD is categorised into quintiles ranging from 1 (most deprived) to 5 (most affluent)[10].

Diagnostic brain scan images and their accompanying reports archived for research purposes will also be accessed for our cohort. By use of each individual's unique Community Health Index (CHI) number, existing diagnostic imaging and radiological reports held within eDRIS will be accessed. We will interrogate routine brain scan images via standardised (semi-)automated processing methods, specifically magnetic resonance imaging (MRI) and computerized tomography (CT) scans, to identify structural brain differences, one example of this being abnormalities of the septum pellucidum. The septum pellucidum is a thin membrane located deep in the centre of the brain, and identification of abnormalities of this structure may be one way in which we can identify neurodegeneration as a result of exposure to brain injury. Assessing white matter structural abnormalities (for example) may aid in establishing a potential diagnostic utility in distinguishing dementia in those with a history of traumatic brain injury, and those with no history of traumatic brain injury.

Patient and Public Involvement

Patients will not be actively involved in this study. However, as the study progresses and data emerge, representative patient and carer organisations, such as the Scottish Acquired Brain Injury Network (SABIN), the charity Headway, and Brain Health Scotland, will be informed of study outcomes via our already developed public engagement pathways. As well as updating these organisations with our findings, we will ask them to promote and share these findings widely within their community.

Statistical Analyses

All statistical analyses will be undertaken on statistical software available in the National SafeHaven. For the majority of analyses, a stratified Cox proportional hazard model will be used to assess the association between TBI and risk of diseases of

For peer review only - http://bmjopen.bmj.com/site/about/guidelines.xhtml

interest – namely neurodegenerative diseases alongside common causes of death for adults living in Scotland, such as ischaemic heart disease, cerebrovascular disease, and respiratory diseases, as well as common mental health outcomes such as depression, anxiety, and alcohol and drug use. A stratified model will be used to take into account the control matched nature of the data.

Strengths and limitations

HEAD-TBI will provide novel insights into the relationship between TBI and neurodegenerative disease risk with potential to inform strategies to identify those at greatest risk of adverse outcome and possibilities for intervention to mitigate risk. In addition, by providing unique opportunity to study trajectory of disease from initiation to outcome, HEAD-TBI will have potential wider application to research in non-TBI associated dementias. It is noted that although we have access to data regarding age, sex, and socioeconomic status of our cohort, we have limited access to information on confounders beyond this. It is known that family history and genetics are non-modifiable risk factors for neurodegenerative diseases, which cannot be taken into account in this study. We also do not have access to any GP notes or any self-report information from participants themselves. Furthermore, the NHS was not founded until 1948, and we therefore may have less complete medical records or no medical records at all for individuals born in the early 1900's. There are also restrictions regarding the diagnostic brain scan images, with data only available between the years 2010-2019.

Ethics and dissemination Approvals for the study have been obtained from the University of Glasgow College of Medical, Veterinary, and Life Sciences Research Ethics Committee (Project Number 200220038) and from National Health Service Scotland's Public Benefits and Privacy Panel (Application 2122-0224). We would anticipate the production of multiple high impact research papers resulting from this week, and we will showcase key results to other researchers in the field, as well as to individuals living with dementia, and care workers in the field of dementia. The interrogation of diagnostic images for abnormalities of brain structure may help us distinguish neurodegenerative diseases resultant from a history of traumatic brain injury and may have diagnostic utility. This work has the potential to inform clinical

For peer review only - http://bmjopen.bmj.com/site/about/guidelines.xhtml

practice, and be of benefit to dementia researchers, clinicians, and individuals suffering with dementia.

For peer review only

For peer review only - http://bmjopen.bmj.com/site/about/guidelines.xhtml

References

1. Alzheimer's Society. How much does dementia care cost? Available at: https://www.alzheimers.org.uk/blog/how-much-does-dementia-care-cost. Accessed: 14/03.22

2. World Health Organisation. Dementia: A Public Health Priority. Available at: https://Apps.Who.Int/Iris/Handle/10665/75263. Accessed: 14/03/22

3. Alzheimer's Disease International. Alzheimer's Innovation Readiness Index.2021. Available at:https://www.alzint.org/resource/alzheimers-innovation-readiness-index-2021. Accessed: 14/03/22

4. Livingston G, Huntley J, Sommerlad A, et al. Dementia prevention, intervention, and care: 2020 report of the Lancet Commission. *Lancet*. 2020. 396(10248):413-446.

5. McMillan TM, McSkimming P, Wainman-Lefley J, et al. Long-Term health outcomes after exposure to repeated concussion in elite level: Rugby union players. *J Neurol Neurosurg Psychiatry*. 2017.88(6):505-511.

6. Wilson L, Stewart W, Dams-O'Connor K, et al. The chronic and evolving neurological consequences of traumatic brain injury. *Lancet Neurol*. 2017. 16:813-825.

7. McMillan TM, Teasdale GM. Death rate is increased for at least 7 years after head injury: A prospective study. *Brain*. 2007. 130:2520-2527.

8. Mackay DF, Russell ER, Stewart K, Maclean J, Pell JP, Stewart W. Neurodegenerative disease mortality among former professional soccer players. *N Engl J Med*. 2019. 381:1801-1801

9. Russell ER, MacKay DF, Stewart K, MacLean JA, Pell JP, Stewart W. Association of Field Position and Career Length with Risk of Neurodegenerative Disease in Male Former Professional Soccer Players. *JAMA Neurol*. 2021. 78(9):1057-1063.

10. SMID. The Scottish Index of Multiple Deprivation. Available at: http://www.gov.scot/Topics/Statistics/SIMD. Accessed: 14/03.22

For peer review only

For peer review only - http://bmjopen.bmj.com/site/about/guidelines.xhtml

Footnotes

Contributors: ERR produced the first draft of the manuscript; DL and WS are involved in supervisory oversight for the HEAD-TBI study, and also co-designed the study protocol and edited the manuscript.

Funding: This study is supported and funded by an early postdoctoral research fellowship from The Chief Scientist Office (CSO), awarded to ERR to undertake this study, starting in October 2022 and concluding in October 2025.

Competing interests: None declared.

Ethics approval: Approval for this study has been provided by the University of Glasgow College of Medical, Veterinary and Life Sciences Ethics Committee (Project Number 200220038). The study has also been approved by National Health Service Scotland's Public Benefit and Privacy Panel (PBPP) for Health and Social Care (Application 2122-0224).

Patient consent for publication: Not required.

For peer review only

For peer review only - http://bmjopen.bmj.com/site/about/guidelines.xhtml

BMJ Open

HEalth And Dementia outcomes following Traumatic Brain Injury (HEAD-TBI): protocol for a retrospective cohort study

Journal:	*BMJ Open*
Manuscript ID	bmjopen-2023-073726.R1
Article Type:	Protocol
Date Submitted by the Author:	18-May-2023
Complete List of Authors:	Russell, Emma; University of Glasgow, School of Psychology and Neuroscience<br>Lyall, Donald; University of Glasgow, School of Health and Wellbeing<br>Stewart, William; University of Glasgow, School of Psychology and Neuroscience ; NHS Greater Glasgow and Clyde, Neuropathology Research Laboratory
**Primary Subject Heading**:	Neurology
Secondary Subject Heading:	Public health, Neurology, Mental health
Keywords:	Dementia < NEUROLOGY, Neuropathology < NEUROLOGY, PUBLIC HEALTH

SCHOLARONE™
Manuscripts

HEalth And Dementia outcomes following Traumatic Brain Injury (HEAD-TBI): protocol for a retrospective cohort study

Emma R Russell[1], Donald M Lyall[2], William Stewart[1,3]

[1]School of Psychology and Neuroscience, University of Glasgow, Glasgow G12 8QQ, UK
[2]School of Health and Wellbeing, University of Glasgow, Glasgow G12 8RZ, UK
[3]Department of Neuropathology, Queen Elizabeth University Hospital, Glasgow G51 4TF, UK

Corresponding Author: Dr Emma Russell, PhD
School of Psychology and Neuroscience
University of Glasgow
Glasgow G12 8QQ, UK

Email: emma.russell@glasgow.ac.uk
Tel: +44(0)141 354 9445

Word Count: 2,952

For peer review only

For peer review only - http://bmjopen.bmj.com/site/about/guidelines.xhtml

Abstract

Background It is estimated that by 2050 the global incidence of dementia will have exceeded 152million. At present there are no effective therapies for dementia, with a focus in research now turning to strategies for disease prevention. Traumatic brain injury (TBI) is recognised as a major risk factor for dementia; estimated to be responsible for at least 3% of cases in the community. However, adverse health outcomes after TBI are not restricted to dementia. A wide range of conditions are documented among TBI survivors, many of which also increase dementia risk. "*HEalth And Dementia outcomes following Traumatic Brain Injury (HEAD-TBI)*" is a study aiming to explore the hypothesis that increased dementia risk following TBI reflects both the direct effect of the injury on the brain and the indirect effects of wider, adverse health outcomes associated with TBI which, in turn, increase dementia risk.

Methods and analysis Comprehensive electronic medical and death certification records will be analysed for individuals with a documented history of TBI, compared with those of a matched general population control cohort with no documented TBI exposure. Cox proportional hazard regression models will be run to compare outcomes. Furthermore, existing diagnostic imaging and radiological reports for the cohort will be analysed to identify evidence of specific white matter abnormalities in TBI exposed individuals and their controls, and establish their potential diagnostic utility.

Ethics and dissemination Approvals for the study have been obtained from the University of Glasgow College of Medical, Veterinary, and Life Sciences Research Ethics Committee (Project Number 200220038) and from National Health Service Scotland's Public Benefits and Privacy Panel (Application 2122-0224). As results emerge, these will be presented at appropriate multi-disciplinary research conferences and made available through open access platforms where possible.

For peer review only - http://bmjopen.bmj.com/site/about/guidelines.xhtml

Strengths and limitations of this study

- Utilising a comprehensive dataset of individuals with a history of traumatic brain injury compared with matched population controls – matched on sex, year of birth, and socioeconomic status – minimises risks of bias.

- It is known that there are limitations in the granularity of the data – brain injury data will be coded in accordance with the International Classification of Diseases, we will not have access to self-reported information or GP/hospital notes.

- Errors in health record coding are widely recognised, but are assumed to be consistent between our cohort of interest (those with a history of traumatic brain injury) and their matched population controls.

- The NHS was not founded until 1948, and we therefore may have less complete medical records for individuals born in the early 1900's.

For peer review only

For peer review only - http://bmjopen.bmj.com/site/about/guidelines.xhtml

Background

Over a quarter of a million people in the UK are living with a dementia diagnosis, with an annual cost to the economy of around £34billion[1]. Despite decades of research, we have no effective therapies for dementia. In part, this is a consequence of the disease having been active for many years by the time a diagnosis is made, by which time the neurodegenerative process is both established and irreversible. Attention, therefore, has begun to focus on developing strategies to prevent or delay the onset of disease. Reflecting this, dementia prevention is regarded as a major public health priority by the World Health Organization[2]. Further, the 2021 Alzheimer's Innovation Readiness Index, co-authored by Alzheimer's Disease International (ADI) and the Global Coalition on Aging (GCOA), calls for national campaigns to address dementia risk factors and for investment in research to better understand their contribution to disease[3].

The 2020 Lancet Commission on Dementia, Intervention and Care identifies 12 potentially modifiable risk factors for dementia[4]. Among these, TBI is estimated to contribute to in excess of 3% of dementias in the community. Importantly, whereas for many, if not all other dementia risk factors, the temporal relationship between risk of exposure and onset of neurodegenerative process is unknown, for TBI the time of injury is known (because it is a discrete, single 'event'). As such, studies in patients with TBI provide unique opportunity to track the development of disease over time, with potential to identify novel strategies for early diagnosis and intervention. However, while the association between TBI and neurodegenerative disease risk is recognised[5–8], this is not the only adverse health outcome associated with TBI[9–11]. In addition to increased risk of neurodegenerative disease, individuals with history of TBI are also recognised as having earlier mortality than expected, with increased risk of many common diseases such as cardiovascular disease, cancer, and respiratory diseases[12]. As such, increased risk of neurodegenerative disease in those surviving TBI might reflect the direct effect of the injury to the brain and/or a consequence of wider adverse health outcomes contributing to increased dementia risk.

HEalth And Dementia outcomes following Traumatic Brain Injury (HEAD-TBI) is specifically designed to assess the complex interaction between TBI and lifelong

For peer review only - http://bmjopen.bmj.com/site/about/guidelines.xhtml

health outcomes contributing to dementia risk following injury. In so doing, HEAD-TBI will provide novel insights into the relationship between TBI and neurodegenerative disease risk with potential to inform strategies to identify those at greatest risk of adverse outcome and possibilities for intervention to mitigate risk. In addition, by providing unique opportunity to study trajectory of disease from initiation to outcome, HEAD-TBI will have potential wider application to research in non-TBI associated dementias. The study will run for a duration of 3 years, during October 2022 to October 2025.

Aims

The specific aims of HEAD-TBI are to:

- explore the risk of dementia and wider neurodegenerative diseases in individuals with a history of TBI compared to matched general population controls.
- compare causes of death and wider health outcomes, including mental health, in individuals with a history of TBI compared to matched general population controls.
- assess the relationship between structural brain imaging differences and risk of dementia following TBI.

Methods and analysis

Using established protocols that have been proven highly successful in addressing similar research aims in other populations of interest[13,14], we now propose conducting a retrospective cohort study looking at a wide range of physical and mental health outcomes, including neurodegenerative disease, in individuals exposed to traumatic brain injury, compared to an age, sex, and social deprivation matched general population comparison group.

Health record interrogation

Individuals with a documented history of TBI will be our cohort of interest, and will be identified from records held by the electronic data research and innovation service (eDRIS), who are part of Public Health Scotland, and hold a wide range of health information, including prescription information and hospital attendance, as well as death certification data for residents in Scotland. Individuals who have prior TBI recorded (coded as international classification of disease 9/10) in their medical records (namely hospital attendance, Scottish Morbidity Record (SMR) 01) will be selected.

For peer review only - http://bmjopen.bmj.com/site/about/guidelines.xhtml

Individuals will be considered to have a documented TBI if their hospital records contain one of the ICD9/ICD10 codes shown in tables 1 & 2. A wide range of TBI severities are listed in the chosen codes, ranging from those with a history of mild TBI such as concussion, to those with more moderate-to-severe brain injuries. This will enable for broad analyses covering all TBI outcomes, to more stratified analyses investigating lifelong health outcomes following certain types or severities of injury. Community health index (CHI) numbers will be used to link individuals to their own electronic health record data. The CHI is a 10-character code, which uniquely identifies each individual in the National Health Service (NHS) Scotland registry.

Table 1: ICD10 codes for traumatic brain injury

S06.0-S06.9	Concussion<br>Traumatic cerebral oedema<br>Diffuse brain injury<br>Focal brain injury<br>Epidural haemorrhage<br>Traumatic subdural haemorrhage<br>Traumatic subarachnoid haemorrhage<br>Intracranial injury with prolonged coma<br>Other intracranial injuries<br>Intracranial injury, unspecified
S07.1, S07.8, S07.9	Crushing injury of skull<br>Crushing injury of other parts of the head<br>Crushing injury of head, unspecified
S09.7-S09.9	Multiple injuries of head<br>Other specified injuries of head<br>Unspecified injury of head

Table 2: ICD9 codes for traumatic brain injury

850.1-850.5, 850.9<br>851.0-854.1	Intracranial injury, including concussion, confusion, laceration, and haemorrhage
959.01	Head injury, unspecified

Matched population controls for the study cohort will be selected on a 3:1 basis, i.e. for every one individual with exposure to traumatic brain injury, three matched controls will be selected. This aims to minimise any bias that may be present, and to increase study power, which can aid the detection of a statistically significant difference in outcome variables. Inclusion criteria for the matched controls will be an absence of any electronic medical history of TBI (any of the ICD9/10 codes for TBI listed in table 1 and 2). This study is specifically designed to look at multiple outcomes emerging

For peer review only - http://bmjopen.bmj.com/site/about/guidelines.xhtml

following TBI and their potential interactions in contributing to neurodegenerative disease risk. As such, the only exclusion in our general population comparison cohort is known TBI history. Exclusion of controls on the basis of epilepsy, by example, risks biasing our data as we would anticipate a proportion of our TBI cohort to develop post-traumatic epilepsy. Similarly, exclusion of controls with history of stroke would bias for vascular risk factors. These matched population controls will also be identified via the electronic Data Research and Innovation Service (eDRIS) of Information Services Division of NHS Scotland. Controls will be matched on year of birth, sex, and socioeconomic status. Socioeconomic status is taken from last known postcode, from which area-based socioeconomic deprivation can be calculated using the Scottish Index of Multiple Deprivation (SIMD) 2020 - which takes into account income, employment, health, education, housing and crime rates of that area. The SIMD is categorised into quintiles ranging from 1 (most deprived) to 5 (most affluent)[15].

Incident neurodegenerative disease diagnoses will be assessed in the cohort. These will be identified from ICD codes from a combination of primary and contributory causes of death as listed on death record certifications, and recorded hospital attendances (Scottish Morbidity Record (SMR) 01), and prescription information. Mental health outcomes will be analysed, identified from mental health hospital admission data (SMR04). The most common causes of death in Scottish males and females will also be assessed. Furthermore, as there are a variety of other known modifiable risk factors for dementia, these will also be assessed in accordance with dementia outcome.

Diagnostic brain scan images and their accompanying reports archived for research purposes will also be accessed for our cohort with a history of TBI, as well as for our control cohort. By use of each individual's unique Community Health Index (CHI) number, existing diagnostic imaging and radiological reports held within eDRIS will be accessed. We will interrogate routine brain scan images via standardised (semi-)automated processing methods, specifically magnetic resonance imaging (MRI) and computerized tomography (CT) scans, to identify structural brain differences in individuals with dementia who have been exposed to TBI with those who have no documented medical history of TBI. One example of a structural brain change which may be seen is abnormalities of the septum pellucidum. The septum pellucidum is a

For peer review only - http://bmjopen.bmj.com/site/about/guidelines.xhtml

thin membrane located deep in the centre of the brain, and identification of abnormalities of this structure may be one way in which we can identify neurodegeneration as a result of exposure to brain injury. Brain scans will be quantitatively assessed (e.g. using FSL) and incorporate multiple brain imaging metrics of relevance to healthy ageing and dementia including hippocampal and associated subcortical phenotypes, total grey/white matter volumes and aspects of cerebrovascular health (e.g. white matter hyperintensities)[16]. Assessing white matter structural abnormalities (for example) may aid in establishing a potential diagnostic utility in distinguishing dementia in those with a history of traumatic brain injury, and those with no history of traumatic brain injury. Regarding availability of suitable imaging studies, the Scottish Medical Imaging Initiative includes diagnostic brain imaging from the years 2010 to 2019. In a previous study of 7,676 former soccer players and their 23,028 matched general population controls the SMI identified images from over 2,000 individuals in the cohort. As such, we are confident there will be adequate imaging studies available for our proposed work.

Prior to the return of the datasets from eDRIS, all individuals in the cohort will be assigned a unique identifier number, which does not enable their identity to be traced. Health record data will be anonymised by removal of name, CHI number, and date of birth from all individuals in the cohort, with date of birth being replaced by month and year of birth only. Access to the excel files containing the datasets will be password protected and accessible via the National SafeHaven by named researchers only.

Patient and Public Involvement

Patients will not be actively involved in this study. However, as the study progresses and data emerge, representative patient and carer organisations, such as the Scottish Acquired Brain Injury Network (SABIN), the charity Headway, and Brain Health Scotland, will be informed of study outcomes via our already developed public engagement pathways. As well as updating these organisations with our findings, we will ask them to promote and share these findings widely within their community.

Statistical Analyses

All statistical analyses will be undertaken on statistical software available in the National SafeHaven. For the majority of analyses, a stratified Cox proportional hazard

For peer review only - http://bmjopen.bmj.com/site/about/guidelines.xhtml

model will be used to assess the association between TBI and risk of diseases of interest – namely neurodegenerative diseases alongside common causes of death for adults living in Scotland, as well as common mental health outcomes. Not only is TBI a risk for neurodegenerative disease, it may also be a consequence of or herald neurodegenerative disease. As such, our data will include time from TBI to measured outcomes, with the time-dependent relationships independently analysed. A stratified model will be used to take into account the control matched nature of the data, whereby we will conduct stratified analyses based on interval. When assessing mortality data only, a competing-risks regression analysis will be undertaken to ascertain whether the estimated hazard ratio is sensitive to the competing risks of other non-neurodegenerative related deaths. Missing data is a commonality in epidemiological research, and we will utilise three fundamental approaches to this: where appropriate, we will acknowledge this as a limitation in each output, impute based on other variables, or replace with mean values. As sensitivity analyses we will do all three and note substantial differences in results.

Strengths and limitations

HEAD-TBI will provide novel insights into the relationship between TBI and neurodegenerative disease risk with potential to inform strategies to identify those at greatest risk of adverse outcome and possibilities for intervention to mitigate risk. Traumatic brain injury (TBI) is recognised as a major risk factor for dementia, however this study will further recognise the importance of greater understanding the other lifelong health outcomes following TBI – many of which may also result in increased risk of dementia. In addition, by providing unique opportunity to study trajectory of disease from initiation to outcome, HEAD-TBI will have potential wider application to research in non-TBI associated dementias.

It is noted that although we have access to data regarding age, sex, and socioeconomic status of our cohort, we have limited access to information on confounders beyond this. It is known that family history and genetics are non-modifiable risk factors for neurodegenerative diseases, which cannot be taken into account in this study. We also do not have access to any GP notes or any self-report information from participants themselves. Furthermore, the NHS was not founded until 1948, and we therefore may have less complete medical records or no medical

For peer review only - http://bmjopen.bmj.com/site/about/guidelines.xhtml

records at all for individuals born in the early 1900's. There are also restrictions regarding the diagnostic brain scan images, with data only available between the years 2010-2019. One limitation is the relative inadequacy of routine electronic health records data to categorise TBI severity. Nevertheless, we will be able to identify individuals with purely mild TBI/ concussion from relevant ICD codes and also to subcategorise for TBI pathologies, such as intracranial hemorrhage, parenchymal injury and diffuse injuries, as surrogates for moderate/severe injury. Furthermore, the health record data only takes into account individuals who have been hospitalised with a traumatic brain injury. This study does not consider patients managed by general practitioners or community based nursing staff, and therefore may not take into consideration patients suffering from concussion / brain injury not requiring secondary care. We further recognise there may be inconsistent reporting of ICD codes however we have no reason to believe there would be any greater inconsistency or systematic bias in recording codes in those who suffered a TBI, and individuals who have not.

Ethics and dissemination Approvals for the study have been obtained from the University of Glasgow College of Medical, Veterinary, and Life Sciences Research Ethics Committee (Project Number 200220038) and from National Health Service Scotland's Public Benefits and Privacy Panel (Application 2122-0224). We would anticipate the production of multiple high impact research papers resulting from this work, and we will showcase key results to other researchers in the field, as well as to individuals living with dementia, and care workers in the field of dementia. The interrogation of diagnostic images for abnormalities of brain structure may help us distinguish neurodegenerative diseases resultant from a history of traumatic brain injury and may have diagnostic utility. This work has the potential to inform clinical practice, and be of benefit to dementia researchers, clinicians, and individuals suffering with dementia.

For peer review only - http://bmjopen.bmj.com/site/about/guidelines.xhtml

References

1. Alzheimer's Society. How much does dementia care cost? Available at: https://www.alzheimers.org.uk/blog/how-much-does-dementia-care-cost. Accessed: 14/03.22

2. World Health Organisation. Dementia: A Public Health Priority. Available at: https://Apps.Who.Int/Iris/Handle/10665/75263. Accessed: 14/03/22

3. Alzheimer's Disease International. Alzheimer's Innovation Readiness Index.2021. Available at:https://www.alzint.org/resource/alzheimers-innovation-readiness-index-2021. Accessed: 14/03/22

4. Livingston G, Huntley J, Sommerlad A, et al. Dementia prevention, intervention, and care: 2020 report of the Lancet Commission. Lancet. 2020. 396(10248):413-446.

5. Lee YK, Hou SW, Lee CC, et al. Increased Risk of Dementia in Patients with Mild Traumatic Brain Injury: A Nationwide Cohort Study. *PLoS One*. 2013;8(5).

6. Gardner RC, Burke JF, Nettiksimmons J, et al. Dementia risk after traumatic brain injury vs nonbrain trauma: The role of age and severity. *JAMA Neurol*. 2014;71(12):1490-1497

7. Schneider ALC, Selvin E, Latour L, et al. Head injury and 25-year risk of dementia. *Alzheimer's and Dementia*. 2021;17(9):1432-1441.

8. Nordström A, Nordström P. Traumatic brain injury and the risk of dementia diagnosis: A nationwide cohort study. *PLoS Med*. 2018;15(1).

9. McMillan TM, McSkimming P, Wainman-Lefley J, et al. Long-Term health outcomes after exposure to repeated concussion in elite level: Rugby union players. J Neurol Neurosurg Psychiatry. 2017.88(6):505-511.

10. Wilson L, Stewart W, Dams-O'Connor K, et al. The chronic and evolving neurological consequences of traumatic brain injury. Lancet Neurol. 2017. 16:813-825.

11. Elser H, Gottesman RF, Walter AE, et al. Head Injury and Long-term Mortality Risk in Community-Dwelling Adults. *JAMA Neurol*. 2023;80(3):260.

12. McMillan TM, Teasdale GM. Death rate is increased for at least 7 years after head injury: A prospective study. Brain. 2007. 130:2520-2527.

13. Mackay DF, Russell ER, Stewart K, et al. Neurodegenerative disease mortality among former professional soccer players. N Engl J Med. 2019. 381:1801-1801

14. Russell ER, MacKay DF, Stewart K, et al. Association of Field Position and Career Length with Risk of Neurodegenerative Disease in Male Former Professional Soccer Players. JAMA Neurol. 2021. 78(9):1057-1063.

15. SMID. The Scottish Index of Multiple Deprivation. Available at: http://www.gov.scot/Topics/Statistics/SIMD. Accessed: 14/03.22

16. Lyall DM, Cox SR, Lyall LM, et al. Association between APOE e4 and white matter hyperintensity volume, but not total brain volume or white matter integrity. *Brain Imaging Behav*. 2020;14(5):1468-1476.

For peer review only - http://bmjopen.bmj.com/site/about/guidelines.xhtml

Footnotes

Contributors: ERR produced the first draft of the manuscript; DL and WS are involved in supervisory oversight for the HEAD-TBI study, and also co-designed the study protocol and edited the manuscript.

Funding: The Chief Scientist Office (CSO), Award Number: EPD/22/07

Competing interests: None declared.

Ethics approval: Approval for this study has been provided by the University of Glasgow College of Medical, Veterinary and Life Sciences Ethics Committee (Project Number 200220038). The study has also been approved by National Health Service Scotland's Public Benefit and Privacy Panel (PBPP) for Health and Social Care (Application 2122-0224).

Patient consent for publication: Not required.

For peer review only

For peer review only

For peer review only - http://bmjopen.bmj.com/site/about/guidelines.xhtml

The RECORD statement – checklist of items, extended from the STROBE statement, that should be reported in observational studies using routinely collected health data.

	Item No.	STROBE items	Location in manuscript where items are reported	RECORD items	Location in manuscript where items are reported
**Title and abstract**					
	1	(a) Indicate the study's design with a commonly used term in the title or the abstract (b) Provide in the abstract an informative and balanced summary of what was done and what was found	Abstract, page 2	RECORD 1.1: The type of data used should be specified in the title or abstract. When possible, the name of the databases used should be included.<br><br>RECORD 1.2: If applicable, the geographic region and timeframe within which the study took place should be reported in the title or abstract.<br><br>RECORD 1.3: If linkage between databases was conducted for the study, this should be clearly stated in the title or abstract.	Abstract, page 2
**Introduction**					
Background rationale	2	Explain the scientific background and rationale for the investigation being reported	Page 4, paragraph 1&2		
Objectives	3	State specific objectives, including any prespecified hypotheses	Page 5, paragraph 1 (Aims)		
**Methods**					

For peer review only - http://bmjopen.bmj.com/site/about/guidelines.xhtml

Study Design	4	Present key elements of study design early in the paper	Page 5, paragraph 2 (Methods and analysis)		
Setting	5	Describe the setting, locations, and relevant dates, including periods of recruitment, exposure, follow-up, and data collection	Page 5, paragraph 3 (Health record interrogation)<br>Page 6, paragraph 2		
Participants	6	*(a) Cohort study* - Give the eligibility criteria, and the sources and methods of selection of participants. Describe methods of follow-up<br>*Case-control study* - Give the eligibility criteria, and the sources and methods of case ascertainment and control selection. Give the rationale for the choice of cases and controls<br>*Cross-sectional study* - Give the eligibility criteria, and the sources and methods of selection of participants	Page 5, paragraph 3; Page 6, paragraph 1&2	RECORD 6.1: The methods of study population selection (such as codes or algorithms used to identify subjects) should be listed in detail. If this is not possible, an explanation should be provided.<br><br>RECORD 6.2: Any validation studies of the codes or algorithms used to select the population should be referenced. If validation was conducted for this study and not published elsewhere, detailed methods and results should be provided.	Page 5, paragraph 3; Page 6, paragraph 1&2<br><br>-
		(b) Cohort study - For matched studies, give matching criteria and number of exposed and unexposed<br>*Case-control study* - For matched studies, give matching criteria and the number of controls per case	Page 6, paragraph 1&2	RECORD 6.3: If the study involved linkage of databases, consider use of a flow diagram or other graphical display to demonstrate the data linkage process, including the number of individuals with linked data at each stage.	-

For peer review only - http://bmjopen.bmj.com/site/about/guidelines.xhtml

Variables	7	Clearly define all outcomes, exposures, predictors, potential confounders, and effect modifiers. Give diagnostic criteria, if applicable.	Page 5, paragraph 3 - (Health record interrogation)	RECORD 7.1: A complete list of codes and algorithms used to classify exposures, outcomes, confounders, and effect modifiers should be provided. If these cannot be reported, an explanation should be provided.	Table 1&2
Data sources/ measurement	8	For each variable of interest, give sources of data and details of methods of assessment (measurement).<br>Describe comparability of assessment methods if there is more than one group			
Bias	9	Describe any efforts to address potential sources of bias	Page 6, paragraph 1&2		
Study size	10	Explain how the study size was arrived at	Page 5, paragraph 3		
Quantitative variables	11	Explain how quantitative variables were handled in the analyses. If applicable, describe which groupings were chosen, and why	-		
Statistical methods	12	(a) Describe all statistical methods, including those used to control for confounding<br>(b) Describe any methods used to examine subgroups and interactions<br>(c) Explain how missing data were addressed	Page 8, paragraph 4 (Statistical analyses)		

For peer review only - http://bmjopen.bmj.com/site/about/guidelines.xhtml

		(d) *Cohort study* - If applicable, explain how loss to follow-up was addressed<br>*Case-control study* - If applicable, explain how matching of cases and controls was addressed<br>*Cross-sectional study* - If applicable, describe analytical methods taking account of sampling strategy<br>(e) Describe any sensitivity analyses			
Data access and cleaning methods		..		RECORD 12.1: Authors should describe the extent to which the investigators had access to the database population used to create the study population.<br><br>RECORD 12.2: Authors should provide information on the data cleaning methods used in the study.	Page 8, paragraph 2
Linkage		..		RECORD 12.3: State whether the study included person-level, institutional-level, or other data linkage across two or more databases. The methods of linkage and methods of linkage quality evaluation should be provided.	Page 8, paragraph 2
**Results**					
Participants	13	(a) Report the numbers of individuals at each stage of the	-	RECORD 13.1: Describe in detail the selection of the persons included in	-

For peer review only

		study (*e.g.*, numbers potentially eligible, examined for eligibility, confirmed eligible, included in the study, completing follow-up, and analysed)<br>(b) Give reasons for non-participation at each stage.<br>(c) Consider use of a flow diagram		the study (*i.e.,* study population selection) including filtering based on data quality, data availability and linkage. The selection of included persons can be described in the text and/or by means of the study flow diagram.	
Descriptive data	14	(a) Give characteristics of study participants (*e.g.*, demographic, clinical, social) and information on exposures and potential confounders<br>(b) Indicate the number of participants with missing data for each variable of interest<br>(c) *Cohort study* - summarise follow-up time (*e.g.*, average and total amount)	-		
Outcome data	15	*Cohort study* - Report numbers of outcome events or summary measures over time<br>*Case-control study* - Report numbers in each exposure category, or summary measures of exposure<br>*Cross-sectional study* - Report numbers of outcome events or summary measures	-		
Main results	16	(a) Give unadjusted estimates and, if applicable, confounder-	-		

For peer review only - http://bmjopen.bmj.com/site/about/guidelines.xhtml

		adjusted estimates and their precision (e.g., 95% confidence interval). Make clear which confounders were adjusted for and why they were included<br>(b) Report category boundaries when continuous variables were categorized<br>(c) If relevant, consider translating estimates of relative risk into absolute risk for a meaningful time period			
Other analyses	17	Report other analyses done—e.g., analyses of subgroups and interactions, and sensitivity analyses	-		
**Discussion**					
Key results	18	Summarise key results with reference to study objectives	-		
Limitations	19	Discuss limitations of the study, taking into account sources of potential bias or imprecision. Discuss both direction and magnitude of any potential bias	Page 9, paragraph 2 (Strengths and limitations)	RECORD 19.1: Discuss the implications of using data that were not created or collected to answer the specific research question(s). Include discussion of misclassification bias, unmeasured confounding, missing data, and changing eligibility over time, as they pertain to the study being reported.	-
Interpretation	20	Give a cautious overall interpretation of results considering objectives, limitations, multiplicity of	-		

For peer review only - http://bmjopen.bmj.com/site/about/guidelines.xhtml

		analyses, results from similar studies, and other relevant evidence			
Generalisability	21	Discuss the generalisability (external validity) of the study results	-		
**Other Information**					
Funding	22	Give the source of funding and the role of the funders for the present study and, if applicable, for the original study on which the present article is based	Page 12 (Funding statement)		
Accessibility of protocol, raw data, and programming code		..		RECORD 22.1: Authors should provide information on how to access any supplemental information such as the study protocol, raw data, or programming code.	-

*Reference: Benchimol EI, Smeeth L, Guttmann A, Harron K, Moher D, Petersen I, Sørensen HT, von Elm E, Langan SM, the RECORD Working Committee. The REporting of studies Conducted using Observational Routinely-collected health Data (RECORD) Statement. *PLoS Medicine* 2015; in press.

*Checklist is protected under Creative Commons Attribution (CC BY) license.

BMJ Open

HEalth And Dementia outcomes following Traumatic Brain Injury (HEAD-TBI): protocol for a retrospective cohort study

Journal:	*BMJ Open*
Manuscript ID	bmjopen-2023-073726.R2
Article Type:	Protocol
Date Submitted by the Author:	20-Jun-2023
Complete List of Authors:	Russell, Emma; University of Glasgow, School of Psychology and Neuroscience<br>Lyall, Donald; University of Glasgow, School of Health and Wellbeing<br>Stewart, William; University of Glasgow, School of Psychology and Neuroscience ; NHS Greater Glasgow and Clyde, Neuropathology Research Laboratory
**Primary Subject Heading**:	Neurology
Secondary Subject Heading:	Public health, Neurology, Mental health
Keywords:	Dementia < NEUROLOGY, Neuropathology < NEUROLOGY, PUBLIC HEALTH

SCHOLARONE™
Manuscripts

HEalth And Dementia outcomes following Traumatic Brain Injury (HEAD-TBI): protocol for a retrospective cohort study

Emma R Russell[1], Donald M Lyall[2], William Stewart[1,3]

[1]School of Psychology and Neuroscience, University of Glasgow, Glasgow G12 8QQ, UK
[2]School of Health and Wellbeing, University of Glasgow, Glasgow G12 8RZ, UK
[3]Department of Neuropathology, Queen Elizabeth University Hospital, Glasgow G51 4TF, UK

Corresponding Author: Dr Emma Russell, PhD
School of Psychology and Neuroscience
University of Glasgow
Glasgow G12 8QQ, UK

Email: emma.russell@glasgow.ac.uk
Tel: +44(0)141 354 9446

Word Count: 3,046

For peer review only - http://bmjopen.bmj.com/site/about/guidelines.xhtml

Abstract

Background It is estimated that by 2050 the global incidence of dementia will have exceeded 152million. At present there are no effective therapies for dementia, with a focus in research now turning to strategies for disease prevention. Traumatic brain injury (TBI) is recognised as a major risk factor for dementia; estimated to be responsible for at least 3% of cases in the community. However, adverse health outcomes after TBI are not restricted to dementia. A wide range of conditions are documented among TBI survivors, many of which also increase dementia risk. "*HEalth And Dementia outcomes following Traumatic Brain Injury (HEAD-TBI)*" is a study aiming to explore the hypothesis that increased dementia risk following TBI reflects both the direct effect of the injury on the brain and the indirect effects of wider, adverse health outcomes associated with TBI which, in turn, increase dementia risk.

Methods and analysis Comprehensive electronic medical and death certification records will be analysed for individuals with a documented history of TBI, compared with those of a matched general population control cohort with no documented TBI exposure. Cox proportional hazard regression models will be run to compare outcomes. Furthermore, existing diagnostic imaging and radiological reports for the cohort will be analysed to identify evidence of specific white matter abnormalities in TBI exposed individuals and their controls, and establish their potential diagnostic utility.

Ethics and dissemination Approvals for the study have been obtained from the University of Glasgow College of Medical, Veterinary, and Life Sciences Research Ethics Committee (Project Number 200220038) and from National Health Service Scotland's Public Benefits and Privacy Panel (Application 2122-0224). As results emerge, these will be presented at appropriate multi-disciplinary research conferences and made available through open access platforms where possible.

For peer review only - http://bmjopen.bmj.com/site/about/guidelines.xhtml

Strengths and limitations of this study

- Utilising a comprehensive dataset of individuals with a history of traumatic brain injury compared with matched population controls – matched on sex, year of birth, and socioeconomic status – minimises risks of bias.

- It is known that there are limitations in the granularity of the data – brain injury data will be coded in accordance with the International Classification of Diseases, we will not have access to self-reported information or GP/hospital notes.

- Errors in health record coding are widely recognised, but are assumed to be consistent between our cohort of interest (those with a history of traumatic brain injury) and their matched population controls.

- The NHS was not founded until 1948, and we therefore may have less complete medical records for individuals born in the early 1900's.

For peer review only

For peer review only - http://bmjopen.bmj.com/site/about/guidelines.xhtml

Background

Over a quarter of a million people in the UK are living with a dementia diagnosis, with an annual cost to the economy of around £34billion[1]. Despite decades of research, we have no effective therapies for dementia. In part, this is a consequence of the disease having been active for many years by the time a diagnosis is made, by which time the neurodegenerative process is both established and irreversible. Attention, therefore, has begun to focus on developing strategies to prevent or delay the onset of disease. Reflecting this, dementia prevention is regarded as a major public health priority by the World Health Organization[2]. Further, the 2021 Alzheimer's Innovation Readiness Index, co-authored by Alzheimer's Disease International (ADI) and the Global Coalition on Aging (GCOA), calls for national campaigns to address dementia risk factors and for investment in research to better understand their contribution to disease[3].

The 2020 Lancet Commission on Dementia, Intervention and Care identifies 12 potentially modifiable risk factors for dementia[4]. Among these, TBI is estimated to contribute to in excess of 3% of dementias in the community. Importantly, whereas for many, if not all other dementia risk factors, the temporal relationship between risk of exposure and onset of neurodegenerative process is unknown, for TBI the time of injury is known (because it is a discrete, single 'event'). As such, studies in patients with TBI provide unique opportunity to track the development of disease over time, with potential to identify novel strategies for early diagnosis and intervention. However, while the association between TBI and neurodegenerative disease risk is recognised[5–8], this is not the only adverse health outcome associated with TBI[9–11]. In addition to increased risk of neurodegenerative disease, individuals with history of TBI are also recognised as having earlier mortality than expected, with increased risk of many common diseases such as cardiovascular disease, cancer, and respiratory diseases[12]. As such, increased risk of neurodegenerative disease in those surviving TBI might reflect the direct effect of the injury to the brain and/or a consequence of wider adverse health outcomes contributing to increased dementia risk.

HEalth And Dementia outcomes following Traumatic Brain Injury (HEAD-TBI) is specifically designed to assess the complex interaction between TBI and lifelong

For peer review only - http://bmjopen.bmj.com/site/about/guidelines.xhtml

health outcomes contributing to dementia risk following injury. In so doing, HEAD-TBI will provide novel insights into the relationship between TBI and neurodegenerative disease risk with potential to inform strategies to identify those at greatest risk of adverse outcome and possibilities for intervention to mitigate risk. In addition, by providing unique opportunity to study trajectory of disease from initiation to outcome, HEAD-TBI will have potential wider application to research in non-TBI associated dementias. The study will run for a duration of 3 years, during October 2022 to October 2025.

Aims

The specific aims of HEAD-TBI are to:

- explore the risk of dementia and wider neurodegenerative diseases in individuals with a history of TBI compared to matched general population controls.
- compare causes of death and wider health outcomes, including mental health, in individuals with a history of TBI compared to matched general population controls.
- assess the relationship between structural brain imaging differences and risk of dementia following TBI.

Methods and analysis

Using established protocols that have been proven highly successful in addressing similar research aims in other populations of interest[13,14], we now propose conducting a retrospective cohort study looking at a wide range of physical and mental health outcomes, including neurodegenerative disease, in individuals exposed to traumatic brain injury, compared to an age, sex, and social deprivation matched general population comparison group.

Health record interrogation

Individuals with a documented history of TBI will be our cohort of interest, and will be identified from records held by the electronic data research and innovation service (eDRIS), who are part of Public Health Scotland, and hold a wide range of health information, including prescription information and hospital attendance, as well as death certification data for residents in Scotland. Individuals who have prior TBI recorded (coded as international classification of disease 9/10) in their medical records (namely hospital attendance, Scottish Morbidity Record (SMR) 01) will be selected.

For peer review only - http://bmjopen.bmj.com/site/about/guidelines.xhtml

Individuals will be considered to have a documented TBI if their hospital records contain one of the ICD9/ICD10 codes shown in tables 1 & 2. A wide range of TBI severities are listed in the chosen codes, ranging from those with a history of mild TBI such as concussion, to those with more moderate-to-severe brain injuries. This will enable for broad analyses covering all TBI outcomes, to more stratified analyses investigating lifelong health outcomes following certain types or severities of injury. Community health index (CHI) numbers will be used to link individuals to their own electronic health record data. The CHI is a 10-character code, which uniquely identifies each individual in the National Health Service (NHS) Scotland registry.

Table 1: ICD10 codes for traumatic brain injury

S06.0-S06.9	Concussion<br>Traumatic cerebral oedema<br>Diffuse brain injury<br>Focal brain injury<br>Epidural haemorrhage<br>Traumatic subdural haemorrhage<br>Traumatic subarachnoid haemorrhage<br>Intracranial injury with prolonged coma<br>Other intracranial injuries<br>Intracranial injury, unspecified
S07.1, S07.8, S07.9	Crushing injury of skull<br>Crushing injury of other parts of the head<br>Crushing injury of head, unspecified
S09.7-S09.9	Multiple injuries of head<br>Other specified injuries of head<br>Unspecified injury of head

Table 2: ICD9 codes for traumatic brain injury

850.1-850.5, 850.9<br>851.0-854.1	Intracranial injury, including concussion, confusion, laceration, and haemorrhage
959.01	Head injury, unspecified

Matched population controls for the study cohort will be selected on a 3:1 basis, i.e. for every one individual with exposure to traumatic brain injury, three matched controls will be selected. This aims to minimise any bias that may be present, and to increase study power, which can aid the detection of a statistically significant difference in outcome variables. Inclusion criteria for the matched controls will be an absence of any electronic medical history of TBI (any of the ICD9/10 codes for TBI listed in table 1 and 2). This study is specifically designed to look at multiple outcomes emerging

For peer review only - http://bmjopen.bmj.com/site/about/guidelines.xhtml

following TBI and their potential interactions in contributing to neurodegenerative disease risk. As such, the only exclusion in our general population comparison cohort is known TBI history. Exclusion of controls on the basis of epilepsy, for example, risks biasing our data as we would anticipate a proportion of our TBI cohort to develop post-traumatic epilepsy. Similarly, exclusion of controls with history of stroke would bias for vascular risk factors. These matched population controls will also be identified via the electronic Data Research and Innovation Service (eDRIS) of Information Services Division of NHS Scotland. Controls will be matched on year of birth, sex, and socioeconomic status. Socioeconomic status is taken from last known postcode, from which area-based socioeconomic deprivation can be calculated using the Scottish Index of Multiple Deprivation (SIMD) 2020 - which takes into account income, employment, health, education, housing and crime rates of that area. The SIMD is categorised into quintiles ranging from 1 (most deprived) to 5 (most affluent)[15].

Incident neurodegenerative disease diagnoses will be assessed in the cohort. These will be identified from ICD codes from a combination of primary and contributory causes of death as listed on death record certifications, and recorded hospital attendances (Scottish Morbidity Record (SMR) 01), and prescription information. Mental health outcomes will be analysed, identified from mental health hospital admission data (SMR04). The most common causes of death in Scottish males and females will also be assessed. Furthermore, as there are a variety of other known modifiable risk factors for dementia, these will also be assessed in accordance with dementia outcome. Known health related modifiable risk factors for dementia may include: hypertension, hearing loss, smoking, obesity, depression, diabetes, and excessive alcohol consumption [4].

Diagnostic brain scan images and their accompanying reports archived for research purposes will also be accessed for our cohort with a history of TBI, as well as for our control cohort. By use of each individual's unique Community Health Index (CHI) number, existing diagnostic imaging and radiological reports held within eDRIS will be accessed. We will be able to match the images back to each individual's health record data, and so can make appropriate statistical adjustments for competing health outcomes. We will control for common psychological (e.g. depression) and cardiometabolic conditions in fully-adjusted models (e.g. coronary heart disease;

For peer review only - http://bmjopen.bmj.com/site/about/guidelines.xhtml

hypertension; diabetes; stroke) based on ICD codes. We will interrogate the routine brain scan images via standardised (semi-)automated processing methods, specifically magnetic resonance imaging (MRI) and computerized tomography (CT) scans, to identify structural brain differences in individuals with dementia who have been exposed to TBI with those who have no documented medical history of TBI. One example of a structural brain change which may be seen is abnormalities of the septum pellucidum. The septum pellucidum is a thin membrane located deep in the centre of the brain, and identification of abnormalities of this structure may be one way in which we can identify neurodegeneration as a result of exposure to brain injury. Brain scans will be quantitatively assessed (e.g. using FSL) and incorporate multiple brain imaging metrics of relevance to healthy ageing and dementia including hippocampal and associated subcortical phenotypes, total grey/white matter volumes and aspects of cerebrovascular health (e.g. white matter hyperintensities)[16]. Assessing white matter structural abnormalities (for example) may aid in establishing a potential diagnostic utility in distinguishing dementia in those with a history of traumatic brain injury, and those with no history of traumatic brain injury. Measures of overall brain age using existing pipelines for research analysis of MRI studies will be employed, comparing TBI to non-TBI individuals, with a main goal of finding an imaging biomarker of adverse brain health after TBI[17,18]. Regarding availability of suitable imaging studies, the Scottish Medical Imaging Initiative includes diagnostic brain imaging from the years 2010 to 2019. In a previous study of 7,676 former soccer players and their 23,028 matched general population controls the SMI identified images from over 2,000 individuals in the cohort. As such, we are confident there will be adequate imaging studies available for our proposed work.

Prior to the return of the datasets from eDRIS, all individuals in the cohort will be assigned a unique identifier number, which does not enable their identity to be traced. Health record data will be anonymised by removal of name, CHI number, and date of birth from all individuals in the cohort, with date of birth being replaced by month and year of birth only. Access to the excel files containing the datasets will be password protected and accessible via the National SafeHaven by named researchers only.

Patient and Public Involvement

For peer review only - http://bmjopen.bmj.com/site/about/guidelines.xhtml

Patients will not be actively involved in this study. However, as the study progresses and data emerge, representative patient and carer organisations, such as the Scottish Acquired Brain Injury Network (SABIN), the charity Headway, and Brain Health Scotland, will be informed of study outcomes via our already developed public engagement pathways. As well as updating these organisations with our findings, we will ask them to promote and share these findings widely within their community.

Statistical Analyses

All statistical analyses will be undertaken on statistical software available in the National SafeHaven. For the majority of analyses, a stratified Cox proportional hazard model will be used to assess the association between TBI and risk of diseases of interest – namely neurodegenerative diseases alongside common causes of death for adults living in Scotland, as well as common mental health outcomes. Not only is TBI a risk for neurodegenerative disease, it may also be a consequence of or herald neurodegenerative disease. As such, our data will include time from TBI to measured outcomes, with the time-dependent relationships independently analysed. A stratified model will be used to take into account the control matched nature of the data, whereby we will conduct stratified analyses based on interval. When assessing mortality data only, a competing-risks regression analysis will be undertaken to ascertain whether the estimated hazard ratio is sensitive to the competing risks of other non-neurodegenerative related deaths. There is a possibility of prodromal cognitive problems prior to any clinical dementia diagnosis, and this may result in some reverse causality, e.g. increased risk of falls. We have access to data regarding time from TBI to dementia outcome, which will, however, be assessed. Missing data is a commonality in epidemiological research, and we will utilise three fundamental approaches to this: where appropriate, we will acknowledge this as a limitation in each output, impute based on other variables, or replace with mean values. As sensitivity analyses we will do all three and note substantial differences in results.

Strengths and limitations

HEAD-TBI will provide novel insights into the relationship between TBI and neurodegenerative disease risk with potential to inform strategies to identify those at greatest risk of adverse outcome and possibilities for intervention to mitigate risk. Traumatic brain injury (TBI) is recognised as a major risk factor for dementia, however

For peer review only - http://bmjopen.bmj.com/site/about/guidelines.xhtml

this study will further recognise the importance of greater understanding the other lifelong health outcomes following TBI – many of which may also result in increased risk of dementia. In addition, by providing unique opportunity to study trajectory of disease from initiation to outcome, HEAD-TBI will have potential wider application to research in non-TBI associated dementias.

It is noted that although we have access to data regarding age, sex, and socioeconomic status of our cohort, we have limited access to information on confounders beyond this. It is known that family history and genetics are non-modifiable risk factors for neurodegenerative diseases, which cannot be taken into account in this study. We also do not have access to any GP notes or any self-report information from participants themselves. Furthermore, the NHS was not founded until 1948, and we therefore may have less complete medical records or no medical records at all for individuals born in the early 1900's. There are also restrictions regarding the diagnostic brain scan images, with data only available between the years 2010-2019. One limitation is the relative inadequacy of routine electronic health records data to categorise TBI severity. Nevertheless, we will be able to identify individuals with purely mild TBI/ concussion from relevant ICD codes and also to subcategorise for TBI pathologies, such as intracranial hemorrhage, parenchymal injury and diffuse injuries, as surrogates for moderate/severe injury. Furthermore, the health record data only takes into account individuals who have been hospitalised with a traumatic brain injury. This study does not consider patients managed by general practitioners or community based nursing staff, and therefore may not take into consideration patients suffering from concussion / brain injury not requiring secondary care. Most likely, mild TBI, mid-moderate TBI, and many psychiatric diagnoses which do not require hospitalization will be overlooked, it is therefore considered that this analysis cannot be generalized to mild TBI and outpatient psychiatric conditions. We further recognise there may be inconsistent reporting of ICD codes however we have no reason to believe there would be any greater inconsistency or systematic bias in recording codes in those who suffered a TBI, and individuals who have not.

Ethics and dissemination Approvals for the study have been obtained from the University of Glasgow College of Medical, Veterinary, and Life Sciences Research Ethics Committee (Project Number 200220038) and from National Health Service

For peer review only - http://bmjopen.bmj.com/site/about/guidelines.xhtml

Scotland's Public Benefits and Privacy Panel (Application 2122-0224). We would anticipate the production of multiple high impact research papers resulting from this work, and we will showcase key results to other researchers in the field, as well as to individuals living with dementia, and care workers in the field of dementia. The interrogation of diagnostic images for abnormalities of brain structure may help us distinguish neurodegenerative diseases resultant from a history of traumatic brain injury and may have diagnostic utility. This work has the potential to inform clinical practice, and be of benefit to dementia researchers, clinicians, and individuals suffering with dementia.

For peer review only

For peer review only - http://bmjopen.bmj.com/site/about/guidelines.xhtml

References

1. Alzheimer's Society. How much does dementia care cost? Available at: https://www.alzheimers.org.uk/blog/how-much-does-dementia-care-cost. Accessed: 14/03.22

2. World Health Organisation. Dementia: A Public Health Priority. Available at: https://Apps.Who.Int/Iris/Handle/10665/75263. Accessed: 14/03/22

3. Alzheimer's Disease International. Alzheimer's Innovation Readiness Index.2021. Available at:https://www.alzint.org/resource/alzheimers-innovation-readiness-index-2021. Accessed: 14/03/22

4. Livingston G, Huntley J, Sommerlad A, et al. Dementia prevention, intervention, and care: 2020 report of the Lancet Commission. Lancet. 2020. 396(10248):413-446.

5. Lee YK, Hou SW, Lee CC, et al. Increased Risk of Dementia in Patients with Mild Traumatic Brain Injury: A Nationwide Cohort Study. *PLoS One*. 2013;8(5).

6. Gardner RC, Burke JF, Nettiksimmons J, et al. Dementia risk after traumatic brain injury vs nonbrain trauma: The role of age and severity. *JAMA Neurol*. 2014;71(12):1490-1497

7. Schneider ALC, Selvin E, Latour L, et al. Head injury and 25-year risk of dementia. *Alzheimer's and Dementia*. 2021;17(9):1432-1441.

8. Nordström A, Nordström P. Traumatic brain injury and the risk of dementia diagnosis: A nationwide cohort study. *PLoS Med*. 2018;15(1).

9. McMillan TM, McSkimming P, Wainman-Lefley J, et al. Long-Term health outcomes after exposure to repeated concussion in elite level: Rugby union players. J Neurol Neurosurg Psychiatry. 2017.88(6):505-511.

10. Wilson L, Stewart W, Dams-O'Connor K, et al. The chronic and evolving neurological consequences of traumatic brain injury. Lancet Neurol. 2017. 16:813-825.

11. Elser H, Gottesman RF, Walter AE, et al. Head Injury and Long-term Mortality Risk in Community-Dwelling Adults. *JAMA Neurol*. 2023;80(3):260.

12. McMillan TM, Teasdale GM. Death rate is increased for at least 7 years after head injury: A prospective study. Brain. 2007. 130:2520-2527.

13. Mackay DF, Russell ER, Stewart K, et al. Neurodegenerative disease mortality among former professional soccer players. N Engl J Med. 2019. 381:1801-1801

14. Russell ER, MacKay DF, Stewart K, et al. Association of Field Position and Career Length with Risk of Neurodegenerative Disease in Male Former Professional Soccer Players. JAMA Neurol. 2021. 78(9):1057-1063.

15. SMID. The Scottish Index of Multiple Deprivation. Available at: http://www.gov.scot/Topics/Statistics/SIMD. Accessed: 14/03.22

16. Lyall DM, Cox SR, Lyall LM, et al. Association between APOE e4 and white matter hyperintensity volume, but not total brain volume or white matter integrity. *Brain Imaging Behav*. 2020;14(5):1468-1476.

17. Spitz G, Hicks AJ, Roberts C, et al.. Brain age in chronic traumatic brain injury. *Neuroimage Clin*. 2022; 35.103039.

For peer review only - http://bmjopen.bmj.com/site/about/guidelines.xhtml

18. Cole JH, Leech R, Sharp DJ. Prediction of brain age suggests accelerated atrophy after traumatic brain injury. *Ann Neurol*. 2015. 77(4), 571–581

For peer review only

For peer review only - http://bmjopen.bmj.com/site/about/guidelines.xhtml

Footnotes

Contributors: ERR produced the first draft of the manuscript; DL and WS are involved in supervisory oversight for the HEAD-TBI study, and also co-designed the study protocol and edited the manuscript.

Funding: The Chief Scientist Office (CSO), Award Number: EPD/22/07

Competing interests: None declared.

Ethics approval: Approval for this study has been provided by the University of Glasgow College of Medical, Veterinary and Life Sciences Ethics Committee (Project Number 200220038). The study has also been approved by National Health Service Scotland's Public Benefit and Privacy Panel (PBPP) for Health and Social Care (Application 2122-0224).

Patient consent for publication: Not required

For peer review only

For peer review only - http://bmjopen.bmj.com/site/about/guidelines.xhtml

For peer review only

For peer review only - http://bmjopen.bmj.com/site/about/guidelines.xhtml

The RECORD statement – checklist of items, extended from the STROBE statement, that should be reported in observational studies using routinely collected health data.

	Item No.	STROBE items	Location in manuscript where items are reported	RECORD items	Location in manuscript where items are reported
**Title and abstract**					
	1	(a) Indicate the study's design with a commonly used term in the title or the abstract (b) Provide in the abstract an informative and balanced summary of what was done and what was found	Abstract, page 2	RECORD 1.1: The type of data used should be specified in the title or abstract. When possible, the name of the databases used should be included.<br><br>RECORD 1.2: If applicable, the geographic region and timeframe within which the study took place should be reported in the title or abstract.<br><br>RECORD 1.3: If linkage between databases was conducted for the study, this should be clearly stated in the title or abstract.	Abstract, page 2
**Introduction**					
Background rationale	2	Explain the scientific background and rationale for the investigation being reported	Page 4, paragraph 1&2		
Objectives	3	State specific objectives, including any prespecified hypotheses	Page 5, paragraph 1 (Aims)		
**Methods**					

For peer review only - http://bmjopen.bmj.com/site/about/guidelines.xhtml

Study Design	4	Present key elements of study design early in the paper	Page 5, paragraph 2 (Methods and analysis)		
Setting	5	Describe the setting, locations, and relevant dates, including periods of recruitment, exposure, follow-up, and data collection	Page 5, paragraph 3 (Health record interrogation)<br>Page 6, paragraph 2		
Participants	6	*(a) Cohort study* - Give the eligibility criteria, and the sources and methods of selection of participants. Describe methods of follow-up<br>*Case-control study* - Give the eligibility criteria, and the sources and methods of case ascertainment and control selection. Give the rationale for the choice of cases and controls<br>*Cross-sectional study* - Give the eligibility criteria, and the sources and methods of selection of participants	Page 5, paragraph 3; Page 6, paragraph 1&2	RECORD 6.1: The methods of study population selection (such as codes or algorithms used to identify subjects) should be listed in detail. If this is not possible, an explanation should be provided.<br><br>RECORD 6.2: Any validation studies of the codes or algorithms used to select the population should be referenced. If validation was conducted for this study and not published elsewhere, detailed methods and results should be provided.	Page 5, paragraph 3; Page 6, paragraph 1&2<br><br>-
		(b) Cohort study - For matched studies, give matching criteria and number of exposed and unexposed<br>*Case-control study* - For matched studies, give matching criteria and the number of controls per case	Page 6, paragraph 1&2	RECORD 6.3: If the study involved linkage of databases, consider use of a flow diagram or other graphical display to demonstrate the data linkage process, including the number of individuals with linked data at each stage.	-

For peer review only - http://bmjopen.bmj.com/site/about/guidelines.xhtml

Variables	7	Clearly define all outcomes, exposures, predictors, potential confounders, and effect modifiers. Give diagnostic criteria, if applicable.	Page 5, paragraph 3 - (Health record interrogation)	RECORD 7.1: A complete list of codes and algorithms used to classify exposures, outcomes, confounders, and effect modifiers should be provided. If these cannot be reported, an explanation should be provided.	Table 1&2
Data sources/ measurement	8	For each variable of interest, give sources of data and details of methods of assessment (measurement).<br>Describe comparability of assessment methods if there is more than one group			
Bias	9	Describe any efforts to address potential sources of bias	Page 6, paragraph 1&2		
Study size	10	Explain how the study size was arrived at	Page 5, paragraph 3		
Quantitative variables	11	Explain how quantitative variables were handled in the analyses. If applicable, describe which groupings were chosen, and why	-		
Statistical methods	12	(a) Describe all statistical methods, including those used to control for confounding<br>(b) Describe any methods used to examine subgroups and interactions<br>(c) Explain how missing data were addressed	Page 9, paragraph 1 (Statistical analyses)		

For peer review only - http://bmjopen.bmj.com/site/about/guidelines.xhtml

		(d) *Cohort study* - If applicable, explain how loss to follow-up was addressed<br>*Case-control study* - If applicable, explain how matching of cases and controls was addressed<br>*Cross-sectional study* - If applicable, describe analytical methods taking account of sampling strategy<br>(e) Describe any sensitivity analyses			
Data access and cleaning methods		..		RECORD 12.1: Authors should describe the extent to which the investigators had access to the database population used to create the study population.<br><br>RECORD 12.2: Authors should provide information on the data cleaning methods used in the study.	Page 8, paragraph 2
Linkage		..		RECORD 12.3: State whether the study included person-level, institutional-level, or other data linkage across two or more databases. The methods of linkage and methods of linkage quality evaluation should be provided.	Page 8, paragraph 2
**Results**					
Participants	13	(a) Report the numbers of individuals at each stage of the	-	RECORD 13.1: Describe in detail the selection of the persons included in	-

For peer review only - http://bmjopen.bmj.com/site/about/guidelines.xhtml

		study (*e.g.*, numbers potentially eligible, examined for eligibility, confirmed eligible, included in the study, completing follow-up, and analysed)<br>(b) Give reasons for non-participation at each stage.<br>(c) Consider use of a flow diagram		the study (*i.e.,* study population selection) including filtering based on data quality, data availability and linkage. The selection of included persons can be described in the text and/or by means of the study flow diagram.	
Descriptive data	14	(a) Give characteristics of study participants (*e.g.*, demographic, clinical, social) and information on exposures and potential confounders<br>(b) Indicate the number of participants with missing data for each variable of interest<br>(c) *Cohort study* - summarise follow-up time (*e.g.*, average and total amount)	-		
Outcome data	15	*Cohort study* - Report numbers of outcome events or summary measures over time<br>*Case-control study* - Report numbers in each exposure category, or summary measures of exposure<br>*Cross-sectional study* - Report numbers of outcome events or summary measures	-		
Main results	16	(a) Give unadjusted estimates and, if applicable, confounder-	-		

For peer review only - http://bmjopen.bmj.com/site/about/guidelines.xhtml

		adjusted estimates and their precision (e.g., 95% confidence interval). Make clear which confounders were adjusted for and why they were included<br>(b) Report category boundaries when continuous variables were categorized<br>(c) If relevant, consider translating estimates of relative risk into absolute risk for a meaningful time period			
Other analyses	17	Report other analyses done—e.g., analyses of subgroups and interactions, and sensitivity analyses	-		
**Discussion**					
Key results	18	Summarise key results with reference to study objectives	-		
Limitations	19	Discuss limitations of the study, taking into account sources of potential bias or imprecision. Discuss both direction and magnitude of any potential bias	Page 9, paragraph 2 (Strengths and limitations)	RECORD 19.1: Discuss the implications of using data that were not created or collected to answer the specific research question(s). Include discussion of misclassification bias, unmeasured confounding, missing data, and changing eligibility over time, as they pertain to the study being reported.	-
Interpretation	20	Give a cautious overall interpretation of results considering objectives, limitations, multiplicity of	-		

For peer review only - http://bmjopen.bmj.com/site/about/guidelines.xhtml

		analyses, results from similar studies, and other relevant evidence			
Generalisability	21	Discuss the generalisability (external validity) of the study results	-		
**Other Information**					
Funding	22	Give the source of funding and the role of the funders for the present study and, if applicable, for the original study on which the present article is based	Page 14 (Funding statement)		
Accessibility of protocol, raw data, and programming code		..		RECORD 22.1: Authors should provide information on how to access any supplemental information such as the study protocol, raw data, or programming code.	-

*Reference: Benchimol EI, Smeeth L, Guttmann A, Harron K, Moher D, Petersen I, Sørensen HT, von Elm E, Langan SM, the RECORD Working Committee. The REporting of studies Conducted using Observational Routinely-collected health Data (RECORD) Statement. *PLoS Medicine* 2015; in press.

*Checklist is protected under Creative Commons Attribution (CC BY) license.

For peer review only - http://bmjopen.bmj.com/site/about/guidelines.xhtml
